# Gender Differences in the Association Between Socioeconomic Status and Cardiometabolic Health: National Health and Nutrition Examination Survey

**DOI:** 10.31586/gjcd.2025.1198

**Published:** 2025-02-17

**Authors:** Shervin Assari, Hossein Zare

**Affiliations:** 1 Department of Internal Medicine, Charles R. Drew University of Medicine and Science, Los Angeles, CA, United States; 2 Department of Family Medicine, Charles R. Drew University of Medicine and Science, Los Angeles, CA, United States; 3 Department of Urban Public Health, Charles R. Drew University of Medicine and Science, Los Angeles, CA, United States; 4 Marginalization-Related Diminished Returns (MDRs) Center, Los Angeles, CA, United States; 5 Department of Health Policy and Management, Johns Hopkins Bloomberg School of Public Health, Baltimore, MD, United States; 6 School of Business, University of Maryland Global Campus (UMGC), Adelphi, MD, United States

**Keywords:** Gender, Sex Differences, Gender Differences, Obesity, Diabetes, Heart Disease, Cardiometabolic Diseases

## Abstract

**Background:**

Socioeconomic status (SES) is a well-established determinant of health, often associated with lower risk of cardiometabolic diseases (CMD). However, the extent to which SES influences CMD may vary by gender due to differences in social roles, health behaviors, and biological susceptibilities. This study examined the relationship between SES, measured by the poverty-to-income ratio (PIR), and CMD indicators—including obesity, diabetes, and cardiovascular disease (CVD)—among men and women using data from the National Health and Nutrition Examination Survey (NHANES).

**Methods:**

This cross-sectional study utilized NHANES data (1999-2018), adjusting for race/ethnicity and age. SES was operationalized using PIR, with CMD outcomes (obesity, diabetes, and CVD) as dependent variables. Generalized linear models (GLM) were employed to evaluate the main effects of SES on CMD, with gender included as a moderator.

**Results:**

Higher SES was associated with lower overall CMD risk. However, the protective effects of SES were more pronounced in women than in men for all outcomes. These findings suggest that gender-specific pathways may mediate the relationship between SES and CMD. Women may derive greater health benefits from higher SES due to factors such as reduced stress exposure, healthier behaviors, and increased healthcare utilization. Conversely, the weaker association observed in men may reflect differences in social hierarchy sensitivity, responses to unemployment, or other contextual factors.

**Conclusion:**

The findings highlight the importance of gender-specific considerations when addressing SES-related disparities in CMD outcomes. Policies and interventions aimed at reducing CMD burden should account for these gender differences to promote equitable improvements in cardiometabolic health. Further research is needed to unravel the mechanisms driving these differences and to inform targeted strategies.

## 1. Introduction

Cardiometabolic diseases (CMD), including obesity, diabetes, and cardiovascular disease (CVD), are leading causes of morbidity and mortality worldwide [1,2]. Socioeconomic status (SES) is a key determinant of CMD, with higher SES often linked to better health outcomes and lower CMD prevalence [3]. This relationship is thought to operate through pathways such as access to healthcare, healthier lifestyles, and reduced exposure to chronic stress [4-7]. However, the influence of SES on CMD may vary by gender, reflecting differences in social roles, stressors, and health behaviors [8,9].

Emerging evidence suggests that men and women differ in the degree and mechanisms through which SES impacts health [10,11]. For men, SES may more strongly influence health through social rank and hierarchy [12], with unemployment and economic instability potentially having more severe consequences [13]. Conversely, women may derive greater benefits from higher SES in the areas of utilization of healthcare, healthier diets, and greater health awareness [14-16]. Additionally, gendered patterns of CMD prevalence—such as higher obesity rates in women [17] and more fatal CVD in men [18]—may complicate the interpretation of SES effects.

While some studies have examined gender differences in the SES-health relationship [10,11], few have focused specifically on CMD outcomes. Moreover, the existing literature often fails to account for the complexity of CMD as a multi-faceted construct encompassing obesity, diabetes, and CVD. Understanding whether SES exerts stronger protective effects on CMD for women than men is crucial for designing effective interventions and policies.

### 1.1. Objectives

The current study aims to explore the main effects of SES, as measured by the poverty-to-income ratio (PIR), on CMD outcomes and to investigate the potential interaction between gender and SES. By using nationally representative data from National Health and Nutrition Examination Survey (NHANES), this study provides valuable insights into the gendered pathways linking SES to CMD. Our findings will contribute to a more nuanced understanding of health disparities and inform targeted strategies for improving CMD outcomes across diverse populations.

## 2. Methods

### 2.1. Design and Setting

This study utilized data from the NHANES 1999-2018 [19], a nationally representative, cross-sectional survey conducted by the Centers for Disease Control and Prevention (CDC). NHANES employs a complex, multistage probability sampling design to assess the health and nutritional status of the noninstitutionalized U.S. population. Data are collected through in-home interviews, physical examinations, and laboratory testing at mobile examination centers. The survey ensures representation of diverse demographic groups through oversampling of certain populations, including racial/ethnic minorities, older adults, and low-income individuals. For this analysis, we focused on participants aged 18 years and older with complete data on socioeconomic status (SES), cardiometabolic disease (CMD) indicators, and demographic variables.

### 2.2. Measures

Socioeconomic status was assessed using the poverty-to-income ratio (PIR), a measure of household income relative to the federal poverty threshold adjusted for family size. PIR values were treated as a continuous variable in the analyses, with higher values indicating higher SES. Cardiometabolic disease outcomes included obesity, diabetes, and cardiovascular disease (CVD). Obesity was defined using body mass index (BMI), with a threshold of 30 kg/m^2^ or higher. CVD outcomes were identified through self-reported history of conditions such as heart attack, coronary heart disease, or stroke. All CMD outcomes were treated as dichotomous variables.

Gender was the primary moderating variable, with analyses stratified by male and female participants to explore gender-specific associations. Covariates included age and race/ethnicity, given their established roles as determinants of CMD. Age was included as a continuous variable, while race/ethnicity was categorized as non-Hispanic White, non-Hispanic Black, Hispanic, and other.

### 2.3. Statistical Analysis

Analyses were conducted using generalized linear models (GLM) [20-23] to evaluate the associations between SES and CMD outcomes, with interaction terms included to assess potential moderation by gender. Sampling weights provided by NHANES were applied to account for the complex survey design and ensure nationally representative estimates. Model 1 included SES as the primary predictor, adjusting for race/ethnicity. Model 2 introduced gender as a moderator by including interaction terms between PIR and gender. All analyses accounted for the dichotomous nature of CMD outcomes, using binomial regression with a logit link function to estimate coefficients (b) and 95% confidence intervals (CIs). The analysis focused on examining both main effects of SES and its interaction with gender on CMD outcomes. Statistical significance was set at p < 0.05. Analyses were performed using Stata software, version 17, incorporating NHANES survey weights and design elements. Results were presented as weighted percentages and adjusted odds ratios to provide an interpretable assessment of SES effects on CMD across genders.

## 3. Results

Table 1 shows the results of the regression analyses examining the associations between socioeconomic status (SES), measured by the poverty-to-income ratio (PIR), and cardiometabolic disease (CMD) outcomes namely diabetes, obesity, and coronary artery disease.

For diabetes, PIR was negatively associated with the outcome in Model 1 (b = −0.371, p < 0.001). In Model 2, the interaction between PIR and female gender was significant (b = −0.434, p < 0.01), indicating that the protective effect of SES was weaker for women compared to men. Additionally, Black participants showed a higher risk of diabetes compared to non-Latino White participants in both models (b = 0.335, p < 0.001). Hispanic individuals and participants from other racial/ethnic groups did not show significant associations with diabetes.

For coronary artery disease (CAD), PIR was negatively associated with CAD in Model 1 (b = −0.496, p < 0.001). The interaction term for PIR and female gender was significant in Model 2 (b = −0.758, p < 0.001), again suggesting that SES had a weaker protective effect for women. Black and Hispanic participants demonstrated a lower risk of CAD compared to non-Latino Whites in both models (Black: b = −0.838, p < 0.001; Hispanic: b = −1.141, p < 0.001 in Model 1).

For obesity, PIR was negatively associated with the outcome in Model 1 (b = −0.104, p < 0.05). The PIR and female gender interaction term was significant in Model 2 (b = −0.539, p < 0.001), indicating that the protective effect of SES on obesity was stronger for women. Black participants were at a higher risk of obesity compared to non-Latino Whites in both models (b = 0.363, p < 0.001 in Model 1), while Hispanic participants demonstrated a lower value of increased risk (b = 0.096, p < 0.05 in Model 1). Female gender was associated with a higher risk of obesity compared to males in both models (b = 0.375, p < 0.001 in Model 2).

Thus, with the exception of HTN, the protective effects of SES (PIR) on CMD outcomes were varied by gender. SES was more strongly protective for men than for women for diabetes, CAD, and obesity.

## 4. Discussion

This study examined the gendered association between socioeconomic status (SES), as measured by the poverty-to-income ratio (PIR), and cardiometabolic disease (CMD) outcomes, including obesity, diabetes, and cardiovascular disease (CVD). The results demonstrated that higher SES is generally associated with lower CMD risk across genders. However, the protective effects of SES on CMD indicators were stronger for women than for men for all CMDs namely diabetes, CAD, and obesity. These findings highlight the necessity of examining gender as a moderating factor in health disparities research and underscore the complexity of SES as a determinant of CMD.

Our results align with existing research that establishes SES as a critical determinant of CMD outcomes [24-29], influencing factors like obesity, diabetes, and CVD. Higher SES likely confers health benefits through access to healthier diets, stress-reducing environments, and quality healthcare. The findings reinforce the need for targeted interventions that allocate resources to low-SES populations, particularly in areas where CMD prevalence is highest. While the benefits of higher SES for CMD prevention were evident for both men and women, women appear to derive greater health benefits, contrary to some prior studies suggesting that SES might have stronger effects on men due to their reliance on social rank and economic stability. This discrepancy could be explained by women’s greater engagement in preventive health behaviors and healthcare utilization.

Chronic stress serves as a crucial mediator in the relationship between SES and CMD, with gender differences playing a significant role in this pathway. Women with higher SES may have different coping mechanisms, resulting in improved health outcomes [30,31]. In contrast, men experience stress differently, particularly in relation to unemployment or perceived economic failure, which may undermine the protective effects of higher SES on CMD outcomes. These differences in stress exposure and coping mechanisms may partially explain the observed gender disparities.

Health behaviors also appear to contribute to the stronger protective effects of SES for women. Women with higher SES are more likely to adopt healthier lifestyles, including regular physical activity, better dietary choices, and avoiding harmful substances like tobacco and alcohol [32-34]. Men, on the other hand, may be less responsive to SES in adopting such behaviors, which could weaken the association between SES and CMD in men [35,36]. This difference in behavioral responses underscores the importance of considering gender in SES-related health interventions.

Healthcare utilization is another critical factor that may explain the gender differences in how SES influences CMD outcomes [37-40]. Higher SES is generally associated with better access to healthcare services, including preventative care. Women tend to be more proactive in seeking medical advice and adhering to treatment recommendations, resulting in better management of conditions like CAD, diabetes, and obesity. Conversely, cultural and societal norms may discourage men from utilizing healthcare services, further contributing to their weaker SES-CMD associations.

The observed gender differences in this study align with a broader body of literature documenting well-established gender disparities in the associations between socioeconomic status (SES), physical health, and mental health. Numerous studies have demonstrated that gender moderates the impact of SES on health outcomes, often revealing differential effects in vulnerability and resilience. These differences may stem from both biological and social mechanisms. Biologically, hormonal and genetic factors can contribute to variations in how men and women experience and respond to stress, illness, and socioeconomic adversity. Socially, gender roles, expectations, and structural inequalities shape access to resources, coping strategies, and exposure to chronic stressors, all of which can influence health trajectories. For example, women may experience heightened psychological distress due to the dual burden of professional and domestic responsibilities, whereas men may encounter societal pressures that discourage help-seeking behaviors, affecting their mental health outcomes differently.

This study specifically examined the intersection of gender and SES, but a more comprehensive intersectional approach would consider additional dimensions of identity and structural disadvantage. Prior research has highlighted that gender does not operate in isolation; rather, its effects on health and socioeconomic outcomes are shaped by intersecting social categories such as race, ethnicity, social environment, and age [43-53]. Intersectionality theory posits that the relationships between these factors are not merely additive but often multiplicative, meaning that the combined impact of multiple marginalized identities can create unique forms of disadvantage or resilience. For instance, the experiences of low-SES Black women may differ significantly from those of low-SES White women or low-SES Black men, reflecting the compounded effects of racial and gender-based discrimination [54-56]. Similarly, older women from disadvantaged backgrounds may face distinct economic and health-related challenges compared to younger women in similar socioeconomic conditions. Future research should incorporate these more nuanced intersectional analyses to better understand the complex ways in which multiple social determinants interact to shape health disparities.

Maharlouei and colleagues conducted a cross-sectional study using data from the General Social Survey (GSS), a nationally representative dataset spanning 1972 to 2018, with a total analytical sample of 65,814 adults. The study examined the associations between educational attainment, employment status, and marital status with self-rated health (SRH) and happiness, both assessed using single-item measures. Age and survey year were included as covariates, while gender served as a moderator. The findings indicated that higher education, employment, and marriage were generally linked to better SRH and happiness. However, gender significantly influenced these relationships—education and marital status had a stronger positive impact on women, whereas employment was more strongly associated with improved outcomes for men. Notably, some inconsistencies emerged when comparing SRH and happiness, suggesting that while these social determinants play a crucial role in subjective well-being, their effects differ by gender [46].

In another study, Assari and colleagues explored the interaction between gender and socioeconomic status (SES) on sleep quality among patients with coronary artery disease (CAD). This cross-sectional analysis included 717 CAD patients and assessed the impact of SES factors—education, income, marital status, and place of residence—on sleep quality, measured using the Pittsburgh Sleep Quality Index (PSQI). Gender was examined as a potential effect modifier, with two-way ANOVA used to test interactions. The results showed that female gender, lower education, and lower income were associated with poorer sleep quality. Specifically, low education and income were predictive of poorer sleep among women but not men, while other SES factors did not exhibit gender-based differences. These findings suggest that among women with CAD, educational and financial resources play a crucial role in sleep quality, whereas these factors appear less relevant for men [47].

Biological differences between men and women add another layer of complexity. For instance, obesity is more prevalent among women, while men are more likely to experience severe and fatal forms of CVD. Additionally, CVD in women often goes undetected or is diagnosed later, which may affect outcome interpretations. These epidemiological and biological variations highlight the importance of tailoring CMD prevention and management strategies to the unique needs of each gender.

### 4.1. Implications

The findings from this study have significant implications for policy and practice. Efforts to address SES-related CMD disparities must incorporate gender-specific considerations to maximize their effectiveness. For women, policies should aim to sustain the protective effects of higher SES by enhancing access to resources, promoting preventive health behaviors, and improving early detection of CMD conditions such as CVD. For men, interventions should focus on mitigating the health impacts of unemployment and economic instability, which are particularly detrimental to their health outcomes. Workplace-based stress reduction programs and accessible mental health services may be effective in addressing these challenges.

### 4.2. Limitations

Despite its contributions, this study has several limitations. The cross-sectional design of NHANES data limits the ability to infer causation between SES, gender, and CMD outcomes. Longitudinal studies are necessary to confirm these findings and explore the temporal dynamics of these relationships. Additionally, while we controlled for race and age, unmeasured confounders such as social support, mental health, and detailed lifestyle behaviors may have influenced the results. Lastly, CMD encompasses a range of conditions with distinct etiologies, and examining these outcomes separately may provide a more nuanced understanding of the SES-CMD relationship.

Future research should focus on addressing these limitations and expanding on the findings. Longitudinal studies are needed to investigate how SES influences CMD trajectories over the life course and how gender moderates these effects. Application of Quantitative Intersectionality Scoring System (QISS) [42] enables researchers to more systematically analyze intersectional effects of various social determinants of health. This system is designed to quantitatively model and capture the complexities of multiple, overlapping social determinants. Studies that use QISS can yield more precise and reliable forecasts for health needs of intersectional populations. Such efforts would allow policymakers and service providers to accurately estimate the health needs of population subgroups. There is a need for more QISS-based models as a response to the complexities related to intersecting identities [42]. Additionally, future work should explore psychosocial factors, workplace dynamics, and healthcare access as mechanisms underlying gender differences in SES-CMD associations. Intersectional factors [57-63], such as race, ethnicity, and cultural norms, should also be examined to provide a more comprehensive understanding of health disparities. Disaggregating CMD outcomes to study the effects of SES on specific conditions like diabetes, obesity, and CVD will further clarify the nuanced pathways linking SES and CMD.

## 5. Conclusion

In conclusion, this study underscores the importance of considering gender in understanding the relationship between SES and CMD outcomes. While higher SES generally protects against CMD, the benefits are stronger for women, reflecting distinct social, behavioral, and biological pathways. Addressing SES-related health disparities requires nuanced strategies that account for these gender differences to achieve equitable improvements in cardiometabolic health. Future research should aim to uncover the mechanisms driving these disparities and inform targeted interventions to reduce CMD risks in diverse populations.

## Figures and Tables

**Table 1.** Coefficient (b) and SE for the associations between SES (PIR) and cardiometabolic disease overall and by gender

	Diabetes			HTN			CAD			Obese	
	Model 1	Model 2		Model 1	Model 2		Model 1	Model 2		Model 1	Model 2
PIR	−0.371 (−0.069)***			−0.043 (−0.115)			−0.496 (−0.101)***			−0.104 (−0.045)*	
Race/Ethnicity											
Non-Hispanic White	Ref	Ref	Ref	Ref	Ref	Ref	Ref	Ref	Ref	Ref	Ref
Non-Hispanic Black	0.335 (−0.071)***	0.335 (−0.071)***		0.181 (−0.115)	0.173 (−0.115)		−0.838 (−0.134)***	−0.826 (−0.14)***		0.363 (−0.049)***	0.347 (−0.049)***
Hispanic	0.112 (−0.077)	0.114 (−0.077)		−0.618 (−0.126)***	−0.626 (−0.127)***		−1.141 (−0.14)***	−1.152 (−0.137)***		0.096 (−0.047)*	0.112 (−0.048)*
3.Other Race/Ethnic Groups	0.225 (−0.111)*	0.243 (−0.111)*		−0.042 (−0.202)	−0.051 (−0.204)		−0.298 (−0.256)	−0.280 (−0.275)		−0.563 (−0.087)***	−0.559 (−0.086)***
											
											
Gender (Female)	−0.134 (−0.063)*	0.080 (−0.073)		0.317 (−0.098)**	0.212 (−0.121)		−0.808 (−0.101)***	−0.426 (−0.116)***		0.104 (−0.045)*	0.375 (−0.050)***
											
High PIR (SES)	Na	−0.151 (−0.098)		Na	−0.132 (−0.146)		Na	−0.102 (−0.103)		Na	0.159 (−0.062)*
PIR x Female	Na	−0.434 (−0.133)**		Na	0.234 (−0.184)		Na	−0.758 (−0.213)***		Na	−0.539 (−0.08)***
											
N	30697	30697		10491	10491		23138	23138		30747	30747

Note: Coefficients are shown outside and SE inside parentheses. * p<0.05, ** p<0.01, *** p<0.001. Sample sizes are different across outcomes due to availability of data on specific outcomes across years.
